# Letter of response to Nabi Z, Stansfeld J, Plöderl M, Wood L, Moncrieff J. Effects of lithium on suicide and suicidal behaviour: a systematic review and meta-analysis of randomised trials. *Epidemiol Psychiatr Sci*. 2022 Sep 16;31:e65. doi: 10.1017/S204579602200049X

**DOI:** 10.1017/S2045796022000671

**Published:** 2022-11-28

**Authors:** T Bschor, M Adli, M Alda, C Baethge, B Etain, T Glenn, P Grof, T Hajek, J Hayes, M Manchia, B Müller-Oerlinghausen, RE Nielsen, P Ritter, JK Rybakowski, G Sani, ML Selo, AH Young, L Tondo

**Affiliations:** Department of Psychiatry, University Hospital Dresden, Fetscherstr. 74, D-01307 Dresden, Germany

In their current meta-analysis, Nabi *et al*. (2022) conclude that there is no convincing evidence that lithium reduces suicide or suicidal behaviour. This conclusion thus contradicts previous high-quality meta-analyses and previous reviews (e.g. Cipriani *et al*., 2013; Smith and Cipriani, 2017; Baldessarini and Tondo 2022).

Since the outcome of a meta-analysis depends on which studies are included (Walker *et al*., 2008), the reasons for the discrepancy between the findings of Nabi *et al*. and previous meta-analyses may depend on the seemingly arbitrary inclusion and exclusion of randomised controlled trials (RCTs) and on an unsatisfactory reinterpretation of data compared to the original publications.

The decision to exclude RCTs published prior to 2000 is puzzling. As a result, more RCTs were excluded (*N* = 15) from the primary analysis than those included (*N* = 12). Also questionable and not justified by the authors is the inclusion of only RCTs comparing lithium against placebo or treatment as usual, but not against an active comparator. At least three studies were excluded on this basis: (1) the randomised comparison of lithium *v.* lamotrigine in bipolar II disorder (Parker *et al*., 2021), (2) the comparison of lithium *v.* valproic acid or carbamazepine in the maintenance therapy of bipolar disorder (Peselow *et al*., 2016) and (3) the comparison of lithium plus quetiapine *v.* quetiapine in the treatment of bipolar depression (AstraZeneca, 2009). Lamotrigine, valproic acid, carbamazepine, and quetiapine are usual treatments for bipolar disorder and we question why these trials were excluded.

Furthermore, the authors state that in a sensitivity analysis they included trials published before 2000, using data extracted in a previous meta-analysis by Cipriani *et al*. (2005). This procedure does not seem accurate, since three key studies from Cipriani *et al.*'s meta-analysis are missing (Greil *et al*., 1996, 1997*a*, 1997*b*).

In addition, the authors' assumption that no suicides took place in studies with no information on suicide events is flawed; this compromises the validity of included data. It also leads to an inflation of zero studies, i.e. studies with no events in either treatment arm, potentially biasing the results towards false negative. Furthermore, the largest RCT included by the authors (Katz *et al*., 2022) reports one death in the lithium group and three deaths in the placebo group. In their meta-analysis however, Nabi *et al*. considered only one suicide per study arm, although the other two deaths were due to an opioid overdose or were classified as suicide by the VA records and the National Death Index (Katz *et al*., 2022), respectively. Accepting three suicides among the placebo-treated participants in Katz *et al*.'s RCT changes the results of the meta-analysis by Nabi *et al*. into a statistically significant anti-suicide effect of lithium (*p* = 0.034, Peto's method).

The analysis of rare events leads to statistical difficulties (Liu, 2019), as discussed in detail in the publication by Nabi *et al*. Nevertheless, the assessment of suicide risk is of the highest clinical relevance. Beyond statistical significance, considering the absolute numbers can therefore help. If in Nabi *et al*.'s meta-analysis waiving the arbitrary criterion of excluding studies before 2000, the 27 RCTs found seven suicides among 1784 subjects in the control arms and only two suicides among the 1953 lithium-treated subjects, a crude reduction of two-thirds (Fig. 3 of Nabi *et al*.'s publication). For the analysis of rare events, it is also useful to use large open studies. In fact, in a meta-analysis of 31 studies with a total of over 85 000 person-years of risk exposure, Baldessarini *et al*. (2006) showed a reduction in suicides and suicide attempts during lithium treatment of four-fifths compared to no lithium therapy (RR = 4.91, 95% CI 3.82–6.31; *p* < 0.0001).

The adequate evaluation of RCTs also provides statistically reliable evidence of the preventive effect of lithium on suicidal acts: In an overview of RCTs, Baldessarini and Tondo (2022) recently found one suicidal act among 622 lithium-treated study participants compared to 14 suicidal acts among 828 study participants in the control groups, resulting in a pooled risk factor of 0.23 (95% CI 0.09–0.59; *p* = 0.002). The meta-analysis by Cipriani *et al*. (2013) found six suicide-related deaths among 241 subjects in the control groups but none among the 244 subjects in the lithium groups (OR 0.13, 95% CI 0.03–0.66; *p* = 0.01). Remarkably, in this meta-analysis, Cipriani *et al*. also demonstrated a statistically significant reduction in all-cause mortality in the lithium groups (OR 0.38, 95% CI 0.15–0.95; *p* = 0.04), thereby confirming older studies (Ahrens *et al*., 1995).

For these reasons, we question the validity of Nabi *et al*.'s findings that are probably flawed and a misleading representation of the evidence base. Looking at the whole picture of all studies available demonstrates the well-established effect of lithium to prevent suicidal acts.
